# Effects of *Vitellaria paradoxa* (C.F. Gaertn.) aqueous leaf extract administration on *Salmonella typhimurium-*infected rats

**DOI:** 10.1186/s12906-017-1643-1

**Published:** 2017-03-21

**Authors:** Siméon Pierre Chegaing Fodouop, Sedric Donald Tala, Lunga Paul Keilah, Norbert Kodjio, Mefokou Didiane Yemele, Armel Herve Nwabo Kamdje, Bridget Nji-kah, Joseph Tchoumboue, Donatien Gatsing

**Affiliations:** 1 Department of Biomedical Sciences, University of Ngaoundéré, PO. Box 454, Ngaoundéré, Cameroon; 2 Laboratory of Microbiology and Antimicrobial Substances, Department of Biochemistry, Faculty of Science, University of Dschang, P.O. Box 67, Dschang, Cameroon; 3 Laboratory of Phytobiochemistry and Medicinal Plants Research, Faculty of Science, University of Yaoundé 1, P.O. Box 812, Yaoundé, Cameroon; 4 Department of Animal Productions, Faculty of Agronomy and Agricultural Sciences, University of Dschang, P.O. Box 222, Dschang, Cameroon

**Keywords:** *Vitellaria paradoxa*, Antimicrobial activity, Side effects, *Salmonellosis*

## Abstract

**Background:**

The present study investigates the effects of *Vitellaria paradoxa* crude extract administration on *Salmonella typhimurium* infected Wistar rats.

**Methods:**

Rats were infected by single dose oral administration of *Salmonella typhimurium* (1.5×10^8^ CFU). Negative control groups were infected and treated orally with distilled water (vehicle), neutral control group were not infected, while the four test groups were treated up to 18 days with 55 mg/kg, 110 mg/kg, 220 mg/kg and 440 mg/kg body weight of aqueous extract of *V. paradoxa* respectively. The effects of this extract administration on serum markers (total protein, creatinine, transaminases, bilirubin and lipid profile) as well as acute toxicity test and phytochemical screening were also investigated.

**Results:**

Following in vivo studies, aqueous extract of *V. paradoxa* allowed to clear salmonellosis in previously infected rats within twelve days of treatment. Infection has resulted in a significant increase of transaminases activity. Besides, significant decrease was observed in liver and kidney relative weight and their protein content. Nevertheless, administration of this plant extract at higher doses has resulted in the correction of some of these injuries. Results obtained from acute toxicity study showed that mice administered with the aqueous leaf extract exhibited a mild reaction to noise and pinch; excreted watery discharges and the LD_50_ value was 12.0 g/kg. In addition, the extract showed no toxic effect after 14 days. However, it may have a sedative effect or depressant effect on the central nervous system, may induce a decrease in plasma levels of algogenic substances, and may cause diarrhea at high doses. Phytochemical screening of the extract revealed the presence of flavonoids, alkaloids, tannins, phenols and polyphenols, saponins, anthocyanins, steroids and anthraquinones.

**Conclusions:**

These results support the ethnomedicinal use of *V. paradoxa,* and suggest that its leave can be used in the management antibacterial phytomedicine.

## Background

*Salmonella enterica* is a group of Gram-negative bacterial pathogens, which cause significant morbidity and mortality worldwide [1]. Among the Salmonellae of medical importance are *Salmonella Typhi*, *Salmonella Paratyphi* A, *Salmonella Paratyphi* B, which cause typhoid, paratyphoid A and paratyphoid B fevers respectively [1]. World-wide, there is an estimated 22 million episodes of typhoid fever causing 216 500 deaths each year, with the overwhelming majority of infections and deaths occurring in developing countries [2].

Even though pharmacological industries produced a number of new antibiotics in the last three decades [3], resistance to these drugs by microorganisms has increased and support the continuous search for alternative drugs [2]. Moreover, chloramphenicol which was used as reference drug against typhoid fever was removed from the market due to inhibition of blood cell production by the bone marrow) [3]. Nature has been a source of medicinal agents for thousands of years, and an impressive number of modern drugs have been isolated from natural sources. This plant-based traditional medicine system has played and continues to play an essential role in health care of many populations. According to World Health Organization, about 80% of the world inhabitants depend mainly on traditional medicines for their primary health care [4, 5]. The utilization of plant extracts in developing countries is not simply a free choice, but is also governed by the growing poverty of people who cannot afford modern medicines.

*V. paradoxa*, commonly known as shea butter tree, belongs to Sapotaceae family and is largely distributed to the semi-arid zone of sub-Saharan Africa from Senegal in the west to Uganda [6]. It is reported to have a great medicinal value particularly in the preparation of skin ointment [7]. Although no scientific work has not yet been done on antibacterial activity of *V. paradoxa* leaves extract, the antibacterial activity of molecules isolated from its root bark have recently been reported [8]. In the West region of Cameroon, leaves and stem bark of *V. paradoxa* are used for the treatment of skin diseases, rheumatism, typhoid fever and microfilaria (in association with other medicinal plants) [9]. Keeping in view the traditional uses and previous work done in vitro on this plant, the present study proposed to investigate the in vivo antibacterial activity of its aqueous leaf extract against *Salmonella typhimurium-*infected rats.

The present study aims at providing to the public a scientific knowledge on the use of *Vitellaria paradoxa* C. F. Gaertn (Sapotaceae), and to look the possible side effects upon short term administration of this extract to mice and infected rats as well as its phytochemistry.

## Methods

### Plant and animal materials

The leaves of *V. paradoxa* were harvested in April 2011 in Fongou village, Noun Division (5° 16 ′ to 5° 35′ north and 10° 30 ′and 10° 45′ east), West Region, Cameroon. The plant was identified at the Cameroon National Herbarium Yaoundé, where a voucher specimen (N°50216/CNH) is deposited. Experimental animals consisted of Swiss Webster mice (CFW®) and Wistar rats (RjHan:WI) were treated in accordance to OECD 2001C/420 [10] and OECD2008a/407 [11] guidelines for Acute Oral Toxicity-fixed dose procedure and for Repeated Dose Oral Toxicity-Rodent respectively.

### Bacteria species

*Salmonella typhimurium* isolate was obtained from the Centre Pasteur of Cameroon, a national public health reference laboratory (Yaoundé, Cameroon).

### Bacterial culture media and preparation of inocula

Tree culture media were used in this work namely Salmonella-Shigella agar (Conda, Madrid, Spain), Mueller Hinton agar (Conda, Madrid, Spain) and Mueller-Hinton broth (MHB) (Conda). The bacterial cell suspensions were prepared at 1.5 × 10^8^ colony-forming units/mL (CFU/ml) following McFarland turbidity standard N° 0.5. For this purpose, 18 h old overnight bacterial cultures were prepared on Mueller-Hinton agar, and few bacteria colonies were collected aseptically with a sterile loop and introduced into 10 ml of sterile 0.90% saline distilled water and homogenised.

### Preparation of the plant extract

*V. paradoxa* leaves were harvested and dried in a ventilated room, at room temperature and ground into powder. The aqueous extract was prepared as described by Alain et al. [12], with some modifications. 500 g of the powder was infused in 2 L of distilled water. The whole was mixed for 2 h at 65 °C using a magnetic stirrer type IKA-MAG RCT. The homogenate obtained was subsequently filtered through Whatman paper filter No. 1, and the extract was obtained by complete evaporation of water in a hot air oven, incubator (memmert) at 50 °C.

### Preparation of the traditional healer therapeutic dose

A handful of fresh leaf of *Vitellaria paradoxa* (sufficient for 1 l of water) was given to us by the traditional healer. This quantity was weighed, and its fresh mass was 400 g. Subsequently, 400 g of *Vitellaria paradoxa* leaves were harvested and prepared as described by the traditional healer. After concentration of the filtrate (approximately 1 L) at 45 °C with a hot air oven, 7.7 g of extract was obtained. According to the traditional healer dose, which is one glass (approximately 300 mL) twice a day, the daily dose was estimated at 55 mg/kg.

### Phytochemical screening

The presence of some secondary metabolites such as alkaloids, steroids, phenols, polyphenols, flavonoids, tannins, saponins, anthraquinones and anthocyanins in the extract were investigated using the methods described by Harbone [13].

### In vivo antimicrobial assay

#### Animal preparation

For this study, 28 mature rats aged between 8 and 9 weeks were used. Prior to their utilization, animals were immunosuppressed by oral administration of 30 mg/kg bw of cyclophosphamide for third day consecutive days as previously described by Abhishek et al. [14].

#### Grouping of animals

Animals were arranged into seven groups of four animals each according to their average body weight. Except group 1 animals which were not infected, the rest were infected by orally administration of 1 ml of a suspension containing 1.5×10^8^ CFU of *S. typhimurium* prepared at 0.5 Mc Farland turbidity scale. The animals were treated as follows:

Group one (neutral control) was not infected and received distilled water during the treatment period.

Group two (negative control group) were infected but were not subsequently treated. Animal of this group received only distilled water during the treatment period.

Group three (positive control) received 5 mg/kg bw of oxytetracyclin [15] during the treatment.

Animals of other groups (four to seven) were treated from the seventh day after infection, with different doses of the plant extract (55, 110, 220 and 440 mg/kg body weight). 55 mg/kg was the traditional healer dose.

During 18 days of treatment, animals were weighed daily and given the treatment before allowing them to feed and drink (tap water) *ad libitum*. The research proposal, conducted according to the Indian government ethical guidelines on the use of animals for scientific research [Committee for Control and Supervision of Experiments on Animals (Registration no. 173/CPCSEA, dated 28 January, 2000)] was approved by the Scientific Postgraduate School board members of the Faculty of Science, University of Dschang, Cameroon.

### Evaluation of biochemical parameters

Prior to sacrifice, animals were subjected to a 12 h food fasting at the end of which urine was collected. After urine collection, animals were anesthetized by injection of Ketamine/Valium (4/1), dissected, followed by a blood collection through a puncture of the abdominal artery and fed into sterilized test tubes. The blood was allowed to stand for 30 min before being centrifuged at 2000 rpm for 10 min using Thermo Labofuge 300 75003230 centrifuge. The sera obtained were used for the determination of biochemical parameters. Organs such as heart, liver, spleen, kidneys and lungs were removed, weighed and their protein content determined.

### Preparation of homogenates

Homogenates of various organs were prepared at 15% (15 g per 100 ml of body) [9], in Tris/HCl Buffer (pH7.2). This was done by grinding 600 mg of organ in 4 ml of buffer. After centrifugation (3000 rpm for 5 min), the supernatant was taken and preserved at −18 °C or immediately used.

### Biochemical analysis

Alanine aminotransferase (ALT) and aspartate aminotransferase (AST) activities were determined using the method proposed by the International Federation of Clinical Chemistry (IFCC) (2002) with Quimica Clinica Aplicada kit, while lipid profile [total cholesterol, triglyceride and high density lipoprotein (HDL)], creatinin and bilirubin were determined by enzymatic colorimetry methods using DIALAB commercial kit. The low density lipoprotein (LDL) was calculated using the formula of Friedewald et al. [16]: LDL = TC - HDL- (TG/5); while atherosclerosis index (LDL/HDL) was calculated using Mertz’s formula [17]. Urinary proteins were measured by the Bradford method (Bradford 1976) [18], while total serum and tissues (liver, heart, spleen and lungs) protein levels were measured by the Biuret method [19]. These parameters were analyzed using UV Shimadzu 120–02 spectrophotometer.

### Histological study

Histological cuts were carried out as described by Mosaid et Alferah [20]. Rats liver and kidneys were removed for histological examination following rat sacrifice. The organs were immediately fixed in 10% neutral buffered formalin, dehydrated with a graded series of ethyl alcohol and embedded in paraffin. Sections (5 mm) were cut and stained with hematoxylin and eosin (Perls’ Prussian blue method). Histological slides were photographed under a Zeiss Axioscope photomicroscope. The reading was done using a light microscope (Olympus) at 100 X magnification. Histopathology was determined based on severity of changes compared to control sections.

### Acute toxicity study

Thirty-five (35) mature mice were divided into 7 groups of 5 animals each. All animals were subjected to 12 h fast prior to administration of the plant extract. Animals in group 2, 3, 4, 5, 6 and 7 were treated with 1 ml of different doses of plant extract *viz.* 2, 4, 8, 16, 18 and 20 g/kg body weight. Group 1 animals (control) received 1 ml of distilled water. Animals of all groups were observed during the first 3 h after a single oral administration of the extract, for behavioural charges. Gathering, locomotion, reaction to noise, reaction to pinch, state of the tail, and appearance of excrement were monitored. When animals are gathered together, it is an indicator of communication (i.e. gathering); they are said to be in activity when they are roaming in the cage; they are say to be reactive when any attempt to touch them, they react by biting. Normal reaction to noise is when the rats are unsettled on hearing a noise; the cries of rats when pinched on their tail is an indicator of normal reaction to pinch; the tail is normal when it is flexible (i.e. no rigid); rigid tail is a sign of anger [21]. After the first 3 h of observation, all animals had free access to food and water. The deaths were counted within the first 48 h; the surviving animals were further observed for two weeks, during which their weights were recorded daily. LD_50_ was determined by calculation using the method of Berhens and Karber [22], as follows:

$$ D{L}_{50}\kern0.5em =\kern0.5em  D{L}_{100}\kern0.5em -\frac{{\displaystyle \sum \left( Z\kern0.5em  x\kern0.5em  D\right)}}{N} $$

DL_100_ = Dose of the substance that causes death of all animals in a test group.

Z = Half of the number of dead animals into two groups corresponding to two consecutive doses.

D = Difference between the doses of two consecutive groups of animals.

N = Number of animal per treated group.

### Statistical analysis

All data were analyzed using one way analysis of variance (ANOVA) followed by post hoc analysis using Waller Duncan test for comparison between treated and control groups [23]. All results were presented as mean ± SEM (standard error of mean). The statistical significance was accepted at *p* < 0.05.

## Results

### Phytochemical tests

Several classes of compounds were identified in the extract of *V. paradoxa* as shown in Table 1. Flavonoids, alkaloids, phenols, polyphenols and cardiac glycosides are present in the aqueous extract. In contrast, triterpens, anthraquinons, anthocyanins, sterols and saponins were not detected.

**Table 1:** Phytochemical composition of extract of *V. paradoxa* leaves. +: Present; − Absent

Class of compound	
Flavonoids	+
Alkaloïds	+
Triterpenoids	-
Phenols	+
Polyphenols	+
Anthraquinones	-
Anthocyanins	-
Sterols	-
Tanins	+
Saponins	-
Cardiac glycosides	+

### In vivo therapeutic properties

**Fig. 1:**
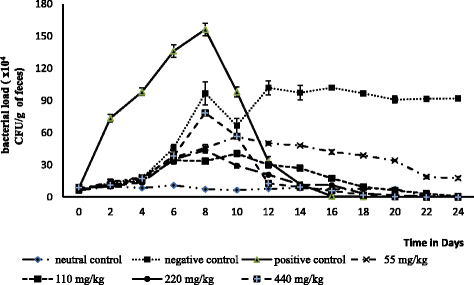
Effect of the V. paradoxa extract on rats Salmonella’s feces load

Figure 1 represents the evolution of the bacterial load (CFU/g of feces) in the feces of experimental rats throughout the experiment. Overall, from the second to the sixth day after infection the bacterial load continuously increased in the feces of infected animals. The figure also shows that from the tenth day (fourth day of treatment), there is a significant and dose-dependent decrease of bacteria load in infected and treated animals. Similarly, there was a slight decrease in bacterial load in negative control group animals which did not receive any treatment, but the load remained relatively high on the last day of treatment (1.93 ×10^6^) as compared to that of animals receiving different doses of extract which were healed after 12 days of treatment.

**Fig. 2:**
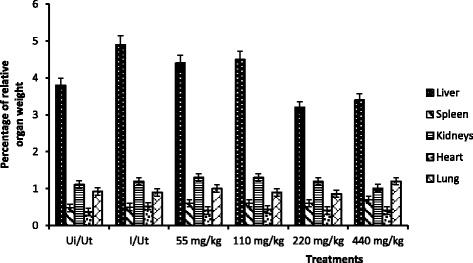
Percentage of organ relative weight. Ui/Ut: Neutral control, I/UT: Negative control. Values are Mean ± SEM of four trials

Figure 2 shows that treatment of animal with different doses of extract does not significantly affect relative weight of kidneys, heart and spleen compared to controls. Also, apart from the significant increases of the spleen relative weight at 440 mg/kg, no other significant chances were observed of spleen’s relative weight as compared to controls. There was a significant infection-related increase in weight of the liver followed by a significant dose-dependent decrease at doses 220 and 440 mg/kg.

**Fig. 3:**
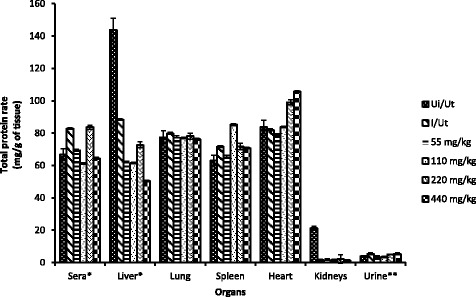
Tissues and urine total protein rate as a function of dose in tested animals. Ui/Ut: neutral control, I/UT: Negative control. Values are Mean ± SEM of four trials. *: Total protein rate in mg/ml. **: normal rate X 10

The protein rate in various tissues of the animals was assessed at the end of the treatment and the results are summarized in Fig. 3. This rate did not change in both spleen and serum. However, the rate of hepatic and renal protein significantly decreased in negative control group animals as compared to the neutral control. This reduction continued dose-dependently in treated groups. In kidneys, renal protein significant increased in animals treated at of 220 mg/kg compared to the negative control. The rate of urinary protein in turn increased significantly in infected and untreated animals and at doses ≥ 220 mg/kg as compared with the neutral control. For heart and spleen, there was a significant increase at ≥ 220 and 440 mg/kg respectively.

**Fig. 4:**
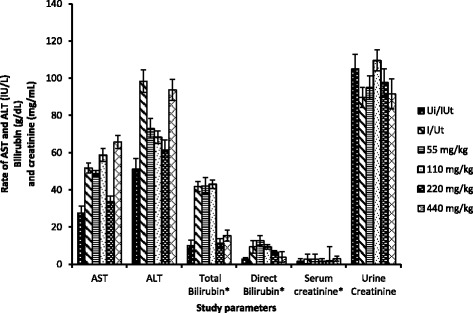
Transaminases, total and direct bilirubin, urinary and serum creatinine as a function of dose in tested animals. Ui/UT: Neutral control, I/Ut: Negative control, *: normal rate X 10. Values are Mean ± SEM of four trials

The levels of circulatory AST and ALT increased significantly (*p* < 0.05) with infection and in treated animals at 440 mg/kg compared to neutral control (Fig. 4). At different doses of treatment, there was a dose-dependent decrease of ALT in infected animals treated with 55 and 110 mg/kg as compared to negative and neutral control. In contrary, AST was not affected at these doses relatively to negative control, but it was significantly increased in all treated group animals as compared to neutral control. It also appeared from this table that the infection resulted in a significant increase of the total and direct bilirubin compared to neutral control returning to normal at the 220 and 440 mg/kg doses. As far as creatinine is concerned, infection of animals with *S. typhimurium* resulted in a significant increase in serum creatinine follow by dose-dependent decreases in groups receiving this extract at doses 55, 110 and 220 mg/kg as compared to neutral control. Beside, urinary creatinine was significantly decreases compare to neutral control but treatment has solved this problem.

### Effect of *V. paradoxa* aqueous extract on lipid profile of rats

**Fig. 5:**
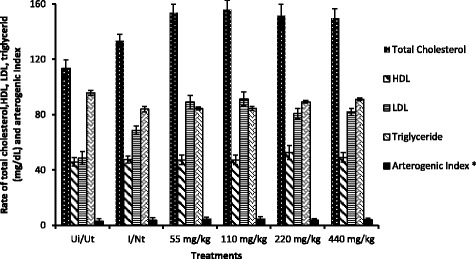
Rate of total cholesterol, HDL (high density lipoprotein), LDL (low density lipoprotein), triglycerides and arteriogenic index of rat after infection and treatment with different doses of *V. paradoxa* aqueous extract. Ui/Ut: Neutral control, I/NT: Negative control. *: twice of the normal value. Values are Mean ± SEM of four trials

Figure 5 shows that the infection resulted in a significant increase of total cholesterol and LDL, and this increase was exacerbated with the administration of different doses of extract. It also shows that the rate of HDL cholesterol, triglycerides and arterogenic index were not significant changed during the treatment, but infection has caused a significant increase in total and LDL cholesterol level. This increase persisted with at different dosess.

### Histological analysis of renal and hepatic cells

**Fig. 6:**
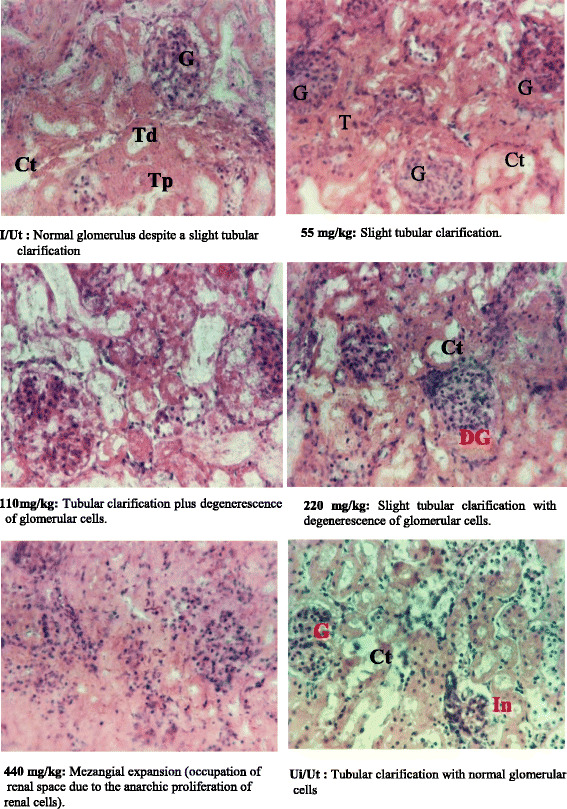
Kidneys photo after histological cut (100x). G = Glomerulus; Td = Distal tubule; Tp = Proximal tubule; Ct = Tubular clarification. DG = Glomerulus degenerescence; I/Ut = negative control; Ui/Ut = neutral control

**Fig. 7:**
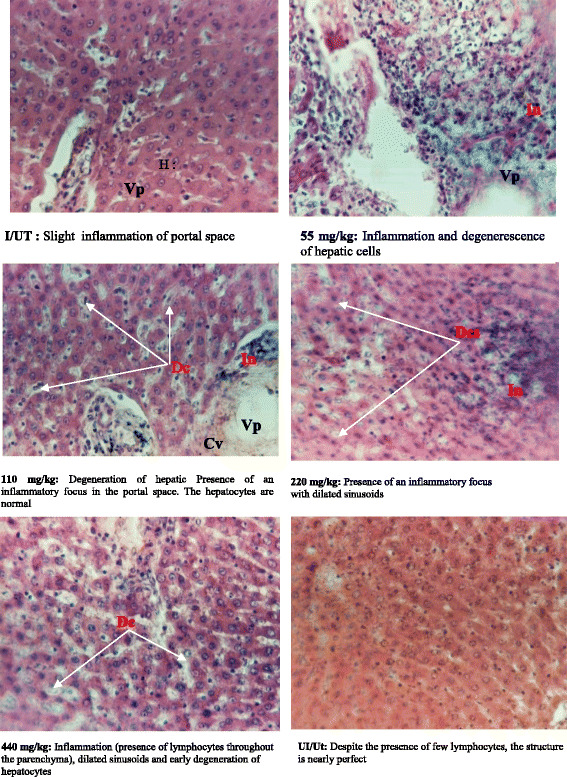
Photos of the liver after histological cut. H = Hepatocytes; Vp = Portal vein; In = Inflammation; Dcs = Dilatation of sinusoids capillaries. Dc = Degenerescence of hepatocytes (characterized by the release of the nuclei of hepatocytes in the sinusoidal space); I/UT = negative control; UI/Ut = neutral control

Figures 6 and 7 below respectively depict the histological cuts of kidney and liver of one animal randomly chosen from each group. It emerges that the infection does not entail any change in appearance of the kidneys since the tubular clarification observed in negative control group are also presented in the neutral control group (uninfected/untraeted). However, at higher doses ≥ 110 mg/kg, there was a dose dependent degeneration of glomerulus, followed by a mezangiale expansion at 440 mg/kg (Fig. 6). Nevertheless Fig. 7 reveals that infection has caused liver modifications which are characterized by the presence of inflammatory foci and degeneration of hepatic cells (release of liver cells nuclei in space sinusoid).

### Acute toxicity test

The behavioural changes observed for gathering, locomotion, reaction to pinch, reaction to noise, state of the tail, consistency of the excrement and the mortality (within 48 h) after the administration of the crude *V. paradoxa* extract at various doses are summarized in Table 2. No behavioural changes were observed in mice receiving the extract at doses ≤ 4 g/kg as compared to the controls. Gathering, locomotion, and state of tail were not affected. On the contrary, there was a decrease in the reaction to noise and reaction to pinch at doses ≥ 16 g/kg. From this same dose, mice’s stools were watery. The animals in all the groups had normal tail (flexible). The mortality was 2, 3, 4 and 5 at doses 8, 16, 18 and 20 g/kg respectively. Based on this mortality, the LD_50_ value was 12.0 g/kg.

**Table 2:** Behavioural changes observed during acute treatment. N: Normal; D: Decrease; P: Pasty; L: Watery; G: Granular

	Doses (g/kg)						
Parameters	0	2	4	8	16	18	20
Gathering	N	N	N	N	N	N	N
Locomotion	N	N	N	N	N	N	N
Reaction to noise	N	N	N	N	D	D	D
Reaction to pinch	N	N	N	N	D	D	D
State of tail	N	N	N	N	N	N	N
Excrement consistency	G	G	G	G	P	W	W
Mortality *within 48 h*	0	0	0	2	3	4	5

## Discussion

### In vivo antisalmonella effect

The establishment of infection was clearly reflected by some changes in animals physiology including the excretion of watery stool, the presence of blood and mucus in the stool, the reduction of activity and the exponential increase in the rate of *S. typhimurium* in the feces of rats after administration of infectious load. This suggested that the bacteria proliferated in the organs after having invaded the system, and challenged the non-specific defense mechanism of rats. The decrease of the bacterial load observed during treatment may be due to the combined action of the extract and immune system given the fact that this decrease was also noted in the negative control group (infected and untreated). Animals treated at therapeutic healing dose (55 mg/kg) recovered in the same period as those treated with multiples of the therapeutic dose. This result suggests that our sample may have a higher activity in vivo. due to their metabolism. The phytochemical screening revealed the presence of several classes of compounds in *V. paradoxa* leaf extract among which: phenols and polyphenols, flavonoids, alkaloids, tannins, saponins, cardiac glycosides and anthocyanins. Some of these secondary metabolites (Flavonoids, alkaloids) have already shown several pharmacological properties including antibacterial properties [24–26]. Since these metabolites are present in our extract, some of them could be associated to the antibacterial activity observed. The increase in total serum cholesterol in negative control group and treated groups might be attributed to the decreased activity of cholesterol 7α-hydroxylase, due to the combined effect of infection and extract administration. This enzyme catalyzes the conversion of cholesterol to bile acid, which is a major route of elimination of cholesterol [27]. This could also be explained by its high production in the liver and its low uptake in the genitals as a precursor of androgen [28]. Infection could have stimulated its hepatic production and the same time inhibited its transfer to genital organs. Despite this increase in total cholesterol level, the extract may have no risk of cardiovascular disease since it did not affect the arterogenic index. Indeed, Jafri et al. [27], has shown a great positive correlation between excess of bad cholesterol (LDL) and/or lack of good cholesterol (HDL) are risk factors for cardiovascular diseases.

Increased serum creatinine followed by a decrease in urinary creatinine observed in infected an none treated group indicates kidney damage [28]. This shows that infection might have result to glomerular alteration, leading to a decrease in its elimination in the urine. This hypotheses could be supported by glomeruloscleroses noted on kidneys histological section. The significant (*p* < 0.05) increase in transaminases in negative control and test groups may be due to animals liver injury [29, 30]. Moreover, animals treated with doses ≥ 220 mg/kg have ALT and AST levels relatively lower than the negative control group (untreated group). These results suggest that at these doses, the extract would induce sudden correction on liver cell damage and would result in an hepatoprotective effect, but significant decreases were observed in liver and kidney relative weight and their protein content, suggesting that this extract might have neither hepatoprotective nor kidneyprotective potential effect. Their protein content could have moved to the blood stream due to the injury of these tissues. This hypothesis can be supported by the presence of flavonoids in our extract. According to Uche et Aprioku [24], flavonoids have anti-inflammatory, antiallergic and hepatoprotective properties. The increase of bilirubin in infected animals could be due to endogenous production of oxidizing substances that have a role in antioxidation (glutathione, uric acid, bilirubin, protein thiol groups, polyamines etc.). The histological sections of liver revealed histopathological changes in infected and untreated animals (negative control) as well as those treated with doses ≥ 220 mg/kg. The presence of inflammatory foci follow by dilation of sinusoids capillaries observed on liver cross sections of test animals might be due to invasion of the liver and destruction of hepatocytes by *salmonella*. At dose 440 mg/kg liver cross section shows a normal appearance, similar to that of neutral group. The extract at this dose would not only treat typhoid, but might had corrected damages caused by the infection.

Kidney cross section shows that apart from a slight tubular clarification observed negative control animals, treatment had resulted in a dose-dependent increase of tubular clarification, glomerular degeneration follow by mezangial espansion in animals treated at a dose 440 mg/ml. This result suggests that, *V. paradoxa* aqueous extract may cause kidney injury. This hypothesis is further supported by the significant increased observed in serum bilirubin and urinary protein.

### Acute toxicity

Overall, acute toxicity study did not reveal any negative behavioural change at lower doses (≤8 g/kg) in mice, as compared to the controls. However, a reduced reaction to noise was observed in mice receiving the extract at doses ≥ 8 g/kg, suggesting that it may have a depressant or sedative effect on the central nervous system [29] at high doses. A reduced reaction to pinch was observed as from 16 g/kg, indicating the effect of the extract on the perception of pain, which may either be due to its nociceptors, to the inhibition of the production of algogenic substances (e.g. prostaglandins, histamines), or to the inhibition of the painful message transmission at the central level [29]. At high doses (≥16 g/kg), the stool was watery, indicating that the extract may cause diarrhoea at these doses. These results suggest that, at higher doses, the extract may have an irritating action on the smooth muscle of the intestinal wall causing a change to fluid and electrolyte permeability [31]. The extract may as well cause an acceleration of the intestinal transit, acting as a laxative [32].

Forty eight hours after administration of different extracts, we found total mortality at the dose 20 g/kg. Convulsion and coma observed few minutes before the death of animals may be due to a decrease in oxygen affinity to hemoglobin because its sites were occupied by the metabolites therein. According to Delongeas et al. [33], the water leaf infusion of *V. paradoxa* can be classified among none toxic substances since the LD_50_ is > 5 g/kg in both sexes.

## Conclusion

Investigations on the aqueous leaf extract of *V. paradoxa* show that this plant contains antimicrobial substances which support its use in local treatment of typhoid fevers. Further work should focus on identification of the active principles of this leaf extract and ascertaining whether they are similar to those described for the stem bark.

## Abbreviations

ALT: Alanine aminotransferase
AST: Aspartate aminotransferase
CFU: Colony forming unit
HDL: High density lipoprotein
LD: Lethal Dose
LDL: Low density lipoprotein
Rpm: Revolutions per minute
SEM: Standard error of mean
