# Correction: Effects of *Vitellaria paradoxa* (C.F. Gaertn.) Aqueous leaf extract administration on *Salmonella typhimurium*-infected rats

**DOI:** 10.1186/s12906-023-03846-8

**Published:** 2023-02-07

**Authors:** Siméon Pierre Chegaing Fodouop, Sedric Donald Tala, Lunga Paul Keilah, Norbert Kodjio, Mefokou Didiane Yemele, Armel Herve Nwabo Kamdje, Bridget Nji-kah, Joseph Tchoumboue, Donatien Gatsing

**Affiliations:** 1 Department of Biomedical Sciences, University of Ngaoundéré, PO. Box 454, Ngaoundéré, Cameroon; 2 Laboratory of Microbiology and Antimicrobial Substances, Department of Biochemistry, Faculty of Science, University of Dschang, P.O. Box 67, Dschang, Cameroon; 3 Laboratory of Phytobiochemistry and Medicinal Plants Research, Faculty of Science, University of Yaoundé 1, P.O. Box 812, Yaoundé, Cameroon; 4 Department of Animal Productions, Faculty of Agronomy and Agricultural Sciences, University of Dschang, P.O. Box 222, Dschang, Cameroon

**Correction: BMC Complement Med Ther 17, 160 (2017)**

**https://doi.org/10.1186/s12906-017-1643-1**

Following publication of the original article [1], the authors identified an error in the author name of Armel Herve Nwabo Kamdje. The correct family name should be Nwabo Kamdje.

The incorrect author name is: Armel Herve kamdje Nwabo

The correct author name is: Armel Herve Nwabo Kamdje

The author group has been updated above and the original article [1] has been corrected.
